# Real-world walking and activity patterns in community-dwelling older adults: a cross-sectional analysis of baseline data from the SMART-AGE trial

**DOI:** 10.1186/s11556-026-00431-z

**Published:** 2026-08-05

**Authors:** Tobias Eckert, Katharina Gordt-Oesterwind, Ann-Sophie Haas, Melissa J. Boettinger, Anna-Lena Schubert, Meike Snijder-Steinhilber, Hans-Werner Wahl, Juergen M. Bauer, Jochen Klenk, Jose Albites–Sanabria, Clemens Becker, Carl-Philipp Jansen

**Affiliations:** 1 Network Aging Research, Heidelberg University, Heidelberg, Germany; 2 Department of Psychology, Johannes Gutenberg University Mainz, Mainz, Germany; 3 Geriatric Center, Medical Faculty Heidelberg, Heidelberg University, Heidelberg, Germany; 4 Robert Bosch Foundation for Medical Research, Stuttgart, Germany; 5 Institute of Epidemiology and Medical Biometry, University of Ulm, Ulm, Germany; 6 Department of Electrical, Electronic, and Information Engineering “Guglielmo Marconi” - DEI, University of Bologna, Bologna, Italy

**Keywords:** Locomotion, Walking, Digital mobility outcomes, Ambulatory monitoring, Wearable electronic devices, Aged

## Abstract

**Background:**

Digital mobility outcomes (DMOs) measured under real-world conditions allow for differentiated insights into mobility patterns of older adults. This study provides an advanced description of real-world walking in community-dwelling older adults and identifies correlates of walking amount, pattern and speed.

**Methods:**

This cross-sectional study used baseline data from the SMART-AGE intervention trial with community-dwelling older adults (age ≥ 67 years). Assessment procedures included one-week monitoring of real-world walking using a wearable device (Axivity AX6), clinical outcomes, and tablet-based questionnaires. DMOs describing the amount (steps, walking duration), pattern (e.g., number of walking bouts [WBs]) and pace (mean and 90th percentile [P90] walking speed) of real-world walking were extracted using a validated computational pipeline. Data were included with ≥ 12 h/day wear time on ≥ 3 days. Stepwise hierarchical linear mixed modelling examined associations between DMOs and sociodemographic correlates, environmental conditions, health status (Body Mass Index), locomotor capacity (Timed Up & Go [TUG]), fall-related concerns (Short FES-I) and cognitive processing speed (Symbol Search Score).

**Results:**

The final sample of 569 participants with a mean age of 75.0 ± 5.6 years (52% women; 56% higher education) showed a mean walking activity of 11 746 ± 5 446 steps/day, 432.3 ± 153.8 WBs / day in total, and a mean walking speed of 0.75 ± 0.13 m/s in longer (> 30s) WBs. Age was consistently related to real-world walking across all DMOs, showing non-linear trends. We found sex-related patterns of walking, as women had more WBs in total but fewer longer (> 30 s) WBs than men. Body Mass Index and locomotor capacity were consistent predictors of walking across all DMOs (β = -0.08 to -0.25, *p* < 0.05).

**Conclusion:**

This study provides detailed and novel insights into real-world walking in a highly active population of older adults, highlighting lower walking activity with increasing age and differences in activity patterns between men and women. Mobility data may serve as a reference for community-dwelling older adults and facilitate the identification of early changes in everyday mobility, thereby informing future mobility recommendations and preventive strategies.

**Supplementary Information:**

The online version contains supplementary material available at 10.1186/s11556-026-00431-z.

## Introduction

Mobility is a major component of healthy ageing and quality of life in older age [1, 2], and its maintenance is associated with better physical and mental health of older adults [3, 4]. Mobility in older age can be described as a multidimensional construct shaped by individual factors (e.g., physical, psychosocial, and cognitive status) as well as environmental and socio-economic factors, consistent with a theoretical framework of mobility in older adults [5]. Although the World Health Organization recommends 150—300 min of moderate or 75—150 min of vigorous physical activity per week [6], only a minority of older adults meet these recommendations. In older adults, walking constitutes a major contributor to overall physical activity and represents the most prevalent form of mobility in old age [7]. Therefore walking-related metrics such as daily step counts are particularly relevant for public health. A recent meta-analysis reported that accumulating at least 6000—8000 steps/day is associated with reduced risk of mortality in older adults [8]. Against this background, assessing walking activity in older adults is essential for identifying potential risk groups and related needs for intervention.

Beyond the amount of walking, the quality-related parameters may provide additional, clinically relevant information. Particularly walking speed is a strong predictor of functional decline and mortality [9]. Traditionally, walking-related characteristics have been assessed under supervised laboratory conditions, referred to as gait [10]. However, walking behavior in daily life differs from supervised gait performance, with older adults typically walking more slowly and less regularly in real-world settings [11, 12]. Real-world assessment is therefore important for capturing mobility as it occurs in everyday life [13].

Wearable sensors enable continuous and objective monitoring of real-world walking [14]. Such real-world assessments have demonstrated added predictive and discriminative power for clinically relevant events [15–17] and health status [18, 19]. While previous studies have mainly focused on the amount of mobility (e.g., steps per day/daily walking time) [20, 21], additional digital mobility outcomes (DMOs) covering other domains of walking, such as the pattern of walking activity (e.g., number of walking bouts) and walking speed, may provide further clinically meaningful insights. For instance, sex-related activity patterns have been reported both in patients recovering from hip fracture [22] and in community-dwelling older adults [23], highlighting the potential need for tailored intervention strategies. However, standardized and validated approaches for assessing these outcomes in older adults remain limited [24].

To bridge this gap, the Mobilise-D consortium developed the MobGap pipeline [25, 26], a validated framework for analyzing real-world walking data in older adults [27, 28]. Applying valid algorithms can provide robust evidence on older adults’ real-world walking performance, facilitating the development of personalized strategies to support active lifestyles and prevent mobility-related decline.

Using this approach, the present study aimed to characterize real-world walking amount, pattern and speed in a sample of older community-dwelling adults. The secondary aim was to examine associations between these real-world mobility metrics and a range of potential correlates, including locomotor capacity, cognition, fall-related concerns, sociodemographic correlates and environmental conditions.

## Methods

### Study design & sample

This observational study is a cross-sectional secondary analysis of baseline data from the SMART-AGE randomized controlled trial, registered at the German Clinical Trials Register (German Clinical Trials Register: DRKS00034316; May 29, 2024). Community-dwelling older adults participated in the SMART-AGE trial, conducted in the cities of Heidelberg and Mannheim, Germany. Both cities are located in the Rhine–Neckar metropolitan region but differ in their sociodemographic and geographic characteristics. Mannheim is an industrial city located entirely in the Upper Rhine Plain, while Heidelberg is a university city at the transition to the Odenwald low mountain range. Participants were recruited between May 2023 and August 2024. Based on city registries, invitation letters with detailed study information were sent to the participants ‘ primary addresses. Participants were included if they were ≥ 67 years old (age of retirement in Germany), lived in their own home environment or assisted living (i.e., independent housing with only minimal support services), were able to speak German, and had no severe physical impairment hampering the participation in a home-based digital exercise program (e.g., Cardiac arrhythmia with dizziness) and/or no cognitive impairment (exclusion criterion: Six Item Cognitive Impairment Test Score ≥ 8, [29]). Detailed information on screening procedure is published elsewhere [30].

### Procedures

A standardized test procedure was conducted in participants’ homes, including clinical outcome assessments (COAs), tablet-based questionnaires, and sensor-based measurements of real-world mobility. COAs and tablet-based questionnaires were assessed using REDCap. Detailed information on recruitment and study procedure is described in the study protocol [30].

#### Real-world walking

Real-world mobility was assessed over a period of one week using a wearable sensor (Axivity AX6, Axivity Ltd, Newcastle Upon Tyne, UK) fixed to the skin of the participants’ lower back using adhesive tape. The device incorporates a 6 Degrees of Freedom inertial measurement unit, including a triaxial accelerometer (range ± 8 g and a resolution of 1 mg), a triaxial gyroscope (2000 degrees per second and a resolution of 70 milli-degrees per second) with a sampling frequency of 100 Hz. We included measurements with a minimum wear time of 3 days for at least 12 h/day [31]. Since the method of fixation does not allow proper re-attachment by the participants themselves, the resulting signal deviation would be detected and flagged by a validated non-wear algorithm [32] applied by the analytical procedure via McRoberts (see below). Using Mobilise-D algorithms, first walking bouts (WBs; defined as walking sequences of at least two consecutive strides of both feet) were identified, allowing for the identification of 6 DMOs on the WB level: duration, number of steps, cadence, walking speed, stride length, and stride duration. Based on that, 24 DMOs were calculated as values on a daily level, differentiating five domains of walking: 1. amount and 2. pattern, 3. pace, 4. rhythm and 5. bout-to-bout variability (Table S1: Overview 24 DMOs). The data processing was conducted via the McRoberts analytical software using the MobGap pipeline v.1 for older adults with no or little mobility restrictions [26].

As a comprehensive analysis of all 24 DMOs provided by the MobGap pipeline is beyond the scope of this manuscript, a subset of six DMOs was selected a priori based on clinical relevance, prior validation evidence, and interpretability. Specifically, these six DMOs have shown evidence of construct validity, including convergent, divergent, and known-groups validity, across two clinical populations [33, 34], and represent key and complementary domains of real-world mobility including amount, pattern, and pace. Walking speed metrics were restricted to longer walking bouts (> 30 s) since these are associated with more reliable and accurate estimates of walking speed according to the Mobilise-D technical validation study [28]. These DMOs were considered to reflect more continuous, steady-state walking under real-world conditions and are likely to capture unrestricted walking outside the home [35], which is particularly relevant for instrumental activities of daily living (e.g., shopping) and leisure-time mobility [36] in independently living older adults, such as those recruited in the SMART-AGE study. In contrast, shorter bouts (10-30s) more often occur in indoor settings and are influenced by starting, stopping, and turning phases, being of higher relevance in individuals with higher mobility restrictions [28].

Therefore, we include the following DMOs in the analyses: 1. number of steps/day (amount), 2. walking duration in minutes/day (amount), 3. number of total WBs/day (pattern), 4. number of longer WBs (> 30 s)/day (pattern), 5. average walking speed in longer WBs (> 30 s) in m/s (pace), and 6. 90th percentile (P90) walking speed in longer (> 30 s) WBs in m/s (pace). P90 was selected to represent near-maximal habitual walking speed under free-living conditions, thereby complementing average walking speed by capturing higher-intensity walking behaviour within daily life.

#### Correlates of real-world walking

The selection of correlates was guided by a theoretical framework of mobility in older adults, distinguishing between sociodemographic, physical, psychosocial, cognitive and environmental correlates [5]. Accordingly, single correlates were selected as representative indicators of these domains for operationalization within the present study. *Sociodemographic correlates* included age, sex, education (as binary variable: higher education defined as university degree or related vs. no university degree) and living status, based on the number of household members (living alone vs not living alone).

As *physical* correlates we assessed various health and function-related outcomes including Body Mass Index (BMI: calculated using the formula ‘weight in kg/height in m2’), comorbidity burden assessed using the Functional Comorbidity Index [37], locomotor capacity measured using the Timed Up and Go test [38]. Cognitive processing speed was assessed using the Symbol Search Test [39], which can be considered a marker of fluid *cognition* [40].

As *psychosocial* construct related to mobility, we assessed fall-related concerns using the Short Falls Efficacy Scale International (Short FES-I) [41]. Life-space mobility was assessed using the Life Space Assessment (LSA) [42]. To avoid redundancy, conceptual circularity, and potential inflation of associations due to overlapping constructs, the LSA was included for descriptive characterization of the study sample only. We used the v0.1 data set for all variables collected in REDCap.

*Environmental conditions* comprised the city of recruitment (Mannheim, Heidelberg) and the season of measurement, operationalized by daylight hours (short day: < 10 h/day; medium day: 10 – 14 h/day; long day: > 14 h/day). Given the short geographical distance between Heidelberg and Mannheim (20 km in total; < 10 km north–south), day length was calculated using the latitude and longitude of Heidelberg and applied to measurements from both cities. For descriptive purposes, we use the three-level categorical variable of season based on daylight hours, while in the analysis for predicting DMOs, we use daylight hours as a metric variable.

### Statistical analyses

Participant characteristics are summarized using means and standard deviations for continuous variables, and frequencies and percentages for categorical variables. Distributional properties of the DMOs were assessed at the measurement-day level using visual inspection of histograms (Figure S1: Histograms) as well as skewness and kurtosis statistics. Based on recommendations skewness values < 2 and kurtosis values < 7 were considered indicative of acceptable deviations from normality [43]. Additionally, residual diagnostics for the final models were performed using Q-Q plots (Figure S2: QQ-Plots). Although these indicated slightly heavier tails than a theoretical normal distribution, no data transformations were applied to maintain the clinical interpretability of the raw units. Descriptive results of all 24 DMOs are presented in Table S2 (Descriptive Results 24 DMOs).

A hierarchical blockwise modeling approach was used to examine associations between digital mobility outcomes (DMOs) and correlates of real-world walking. Potential predictors were selected a priori based on a socioecological framework of mobility in older adults to ensure theoretical coverage of key mobility-related domains. The hierarchical modeling strategy was chosen to derive parsimonious and interpretable models while avoiding overparameterization given the number of investigated DMOs and potentially correlated predictors. Separate linear mixed-effects models (LMMs) were fitted for each DMO (Table 1), including participant and weekday as random intercepts to account for repeated daily measurements and residual day-specific variability.

**Table 1:** Hierarchical blockwise linear mixed modeling approach for predicting digital mobility outcomes (DMOs)

Model Stage	Fixed Effects	Random Effects	Purpose
Base Model	Age, sex, educational level, living status, city, daylight hours and working day-weekend	Participant, Weekday	evaluate basic sociodemographic and environmental variables
Quadratic Age term Model	Step 1 + quadratic age term	Participant, Weekday	Test non-linear age effects
Final Model	Best fitting model (1 or 2) + BMI, FCI, TUG, Short FES-I, symbols search score	Participant, Weekday	Evaluate potential modifiable correlates related to health status, locomotor capacity, fall-related concerns and cognitive function

*Abbreviations*: *BMI* Body mass index, *FCI* Functional Comorbidity Index, *TUG* Timed Up and Go test, *Short FES-I* Falls Efficacy Scale–International Short Version

In the base model, sociodemographic and environmental variables including a working day–weekend indicator (working day: Monday-Friday, weekend: Saturday-Sunday) were entered as fixed effects. The working day-weekend indicator was modelled as fixed effect because the analysis aimed to estimate systematic population-level differences between weekdays and weekends. All dichotomous predictors were effect-coded using sum contrasts (−1/+ 1) prior to model estimation. A quadratic age term was subsequently added to test for non-linear age effects, with model fit compared using likelihood-ratio tests as well as Akaike and Bayesian Information Criteria (AIC, BIC). The best-fitting age specification was retained.

In the final model, potentially modifiable individual characteristics related to health status, locomotor capacity, fall-related concerns, and cognitive function were added while adjusting for significant (*p* ≤ 0.05) sociodemographic and environmental covariates identified in earlier steps.

All analyses were conducted using a complete-case approach without imputation*.* At each model stage, p-values for fixed effects were adjusted using the Benjamini–Hochberg false discovery rate procedure within each mobility domain (amount, pattern, and pace), grouping related DMOs per domain. For each DMO, we report model fit by using marginal R^2^_m_ (variance explained by fixed effects), conditional R^2^c (variance explained by both fixed and random effects), and the intraclass correlation coefficient (ICC; proportion of total variance attributable to between-participant differences), and standardized regression coefficients (Beta). To ensure comparability across DMOs, ICCs were calculated from unconditional random-intercept models including participant-level clustering only. Model fit indices are reported as decimal values, and standardized regression coefficients (Beta) are presented for all fixed effects. Regression coefficients represent deviations from the grand mean rather than direct group comparisons. Because the final adjustment sets differed across DMOs following the hierarchical model selection procedure, standardized regression coefficients were interpreted within each DMO only and were not intended for direct comparison across different DMOs. Statistical analyses were performed using RStudio 4.4.3 [44].

No a priori sample size calculation was conducted for the present secondary cross-sectional analyses, the sample size was determined based on the primary outcomes of the randomized controlled trial Post-hoc sensitivity analyses indicated that the available sample size provided a statistical power of > 0.99 to detect medium-sized effects (f^2^ = 0.15). For small effects (f^2^ = 0.02) statistical power ranged from 0.60 to 0.71 depending on the number of predictors included in the models.

## Results

### Sample description

A total of 649 study partners were included in the SMART-AGE trial. For the present study, data from 569 individuals with valid mobility measurements were eligible, resulting in a mean of 6.2 ± 0.8 days of mobility measured per individual. Details on sample attrition (*n* = 80) are provided in the Supplementary Material (Table S3: Reasons for exclusion). The sample included in our analyses had a mean age of 75.0 ± 5.6 years, consisted of 291 women (52%), and had a high locomotor capacity and spatial mobility (Table 2). Of the total 3 404 valid days of measurement, 37% were performed during summer days with more than ≥ 14 daylight hours.

**Table 2:** Sample characteristics, stratified by the completion of sensor-based activity monitoring

Variable	Total (*N* = 649)	Participants with valid sensor-based measurements (*n* = 569)	Participants without valid sensor-based measurement (*n *= 80)
Age, years, mean (SD)	75.0 (5.6), *n* = 649	75.0 (5.6), *n* = 569	75.4 (6.0), *n* = 80
Sex, female, n (%)	336 (52%), n = 649	291 (51%), n = 569	45 (56%), n = 80
Living status, living alone, n (%)	223 (37%), n = 606	195 (37%), n = 531	28 (37%), n = 75
Education higher (university degree), n (%)	348 (56%), n = 620	303 (56%), n = 543	45 (58%), n = 77
City, *n* (%)			
Heidelberg	360 (55%), *n* = 649	313 (55%), *n* = 569	47 (59%), *n* = 80
Mannheim	289 (45%), *n* = 649	256 (45%), *n* = 569	33 (41%), *n* = 80
Season of Recruitment, number of days with			
daylight < 10 h	––	n = 976 (29%), n = 3,404	–-
10–14 h		n = 1159 (34%), n = 3,404	
> 14 h		n = 1269 (37%), n = 3,404	
BMI, kg/m², mean (SD)	26.6 (4.4), n = 645	26.6 (4.5), n = 567	26.5 (3.8), n = 78
Functional Comorbidity Index, mean (SD)	2.1 (1.7), n = 639	2.1 (1.7), n = 561	2.4 (1.9), n = 78
Timed Up & Go Test, s, mean (SD)	9.8 (2.6), n = 644	9.8 (2.7), n = 566	9.6 (2.1), n = 78
Life Space Assessment (0–120), mean (SD)	87.1 (14.0), n = 576	87.3 (14.1), n = 511	86.2 (12.8), n = 65
Short-FES-I (7–28), mean (SD)	8.1 (1.6), n = 610	8.1 (1.6), n = 540	8.2 (1.6), n = 70
Symbol Search Score, mean (SD)	22.0 (6.5), n = 648	22.0 (6.4), n = 569	21.9 (7.3), n = 79

Presented are participants’ characteristics at baseline of the SMART-AGE intervention trial including sociodemographic correlates, environmental conditions, locomotor capacity, health status, cognitive function, subjective mobility and fall-related concerns for the total sample, participants with and without valid sensor-based measurements

*Abbreviations*: *BMI* Body Mass Index, *Short FES-I* Short Falls Efficacy Scale International

Missing data rates were low across all study variables. No missing values occurred for walking duration, step count, or walking bout frequency measures. Only 12 days (< 0.4%) lacked sufficient WB for the calculation of mean and P90 walking speed in longer (> 30 s) WB. Detailed information is provided in the Supplementary Material (Table S4: Missing data).

### Associations with sociodemographic correlates

**Fig. 1:**
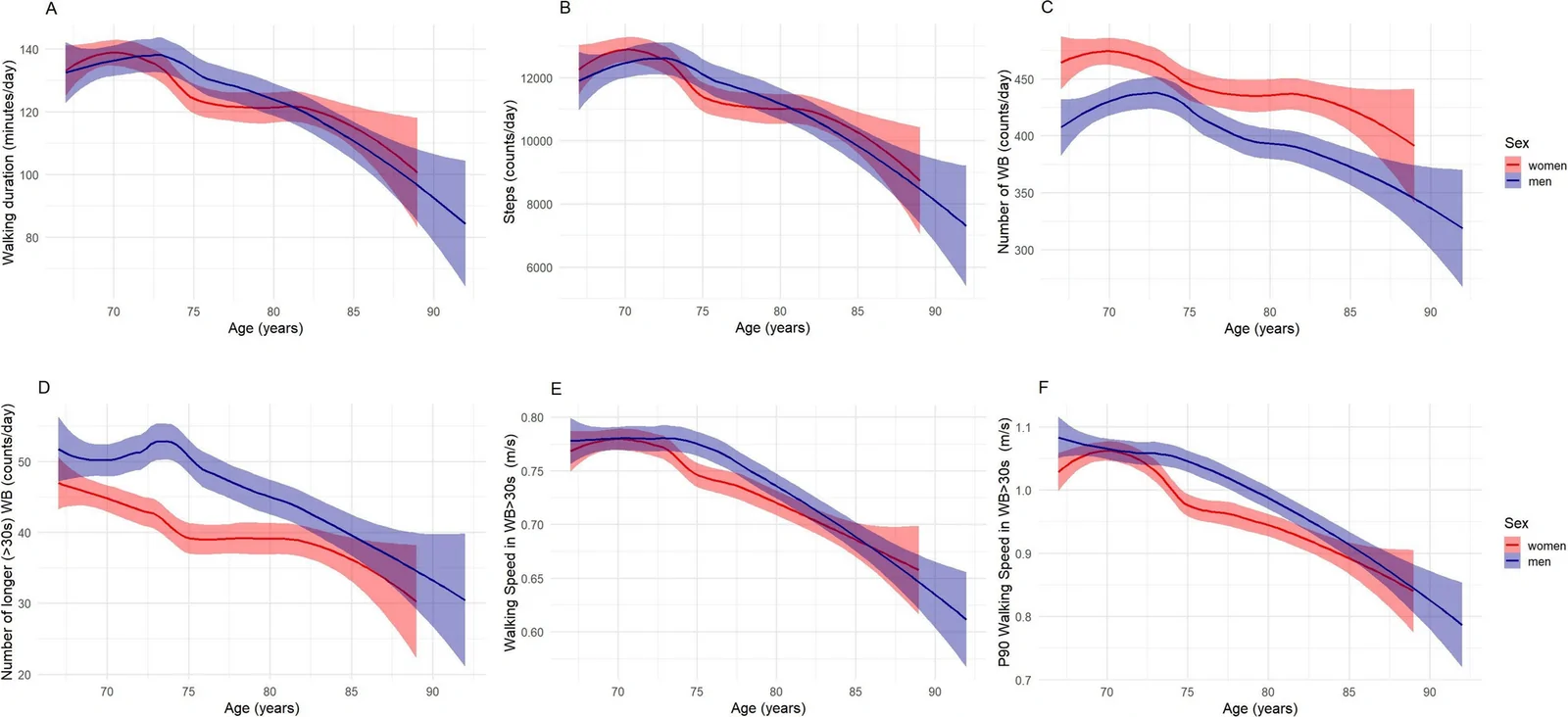
Age-related changes of Digital Mobility Outcomes (DMOs) stratified by sex. Presented are trend lines showing the median and the 25th and 75th percentiles of: **A** Total walking duration in minutes/day; **B** Number of steps/day; **C** Number of total walking bouts/day; **D** Number of longer (> 30 s) walking bouts/day; **E** Mean walking speed in longer (> 30 s) walking bouts; **F** P90 walking speed in longer (> 30 s) walking bouts. Notes: For visualization purposes, unadjusted trend lines were used to illustrate the relationship between age and DMOs stratified by sex. As living status, weekday–weekend differences, city of residence, and daylight duration did not vary systematically by age or sex, adjusted model-based curves were not shown to preserve interpretability

Descriptive visual inspection of the trendline plots, based on raw data, suggests a non-linear association between age and real-world walking across all DMOs, characterized by a plateau phase reached at 75 years of age and a subsequent steady decline in both sexes (Fig. 1).

Consistent with the patterns observed in the descriptive trendline plots, the base LMMs showed significant negative associations between age and all six DMOs (all *p* ≤ 0.01), with standardized small to moderate effect sizes (β = −0.10 to −0.26) (Table S5: Fixed Effects Step1).

Quadratic age terms were subsequently tested to assess non-linear effects (Table S6: Model fit comparison quadratic age effects). Model comparisons indicated no consistent improvement in model fit for the DMOs related to amount and pattern. In contrast, improved model fits were observed for mean and P90 walking speed, as evidenced by significant likelihood-ratio tests (mean walking speed: χ^2^ = 6.0 *p* = 0.014; P90 walking speed: χ^2^ = 4.1, *p* = 0.043) and lower AIC values (ΔAIC = − 4.0 and − 2.1 respectively), while BIC values favored the simpler linear models (ΔBIC = 2.1 and 4.0). Accordingly, quadratic age terms were retained only for speed-related outcomes.

Both the descriptive trendline plots and the results shown in Table 3 indicated potential sex-related differences in activity patterns. Specifically, women demonstrated a greater total number of WB (women, mean ± SD: 453.1 ± 152.0 vs. men: 410.9 ± 152.5), whereas men showed a higher prevalence of longer (> 30 s) WB (women: 41.5 ± 24.3 vs men: 47.7 ± 27.9). The base LMMs confirmed these descriptive patterns, showing significant small-to-moderate associations of sex with the DMOs related to pattern (all p < 0.05). In addition, men showed significantly higher P90 walking speed (β = 0.20, *p* = 0.012), whereas no significant sex differences were observed for mean walking speed during longer-duration (> 30 s) WB (β = 0.12, *p* = 0.093).

**Table 3:** Descriptive statistics of digital mobility outcomes stratified by sex, education, and weekday/weekend

*Digital Mobility Outcome (DMO)*	*Total* *mean (SD)*	*Sex*		*Education*		*Weekday vs. Weekend*	
		*Female* *mean (SD)*	*Male* *mean (SD)*	*High (university degree)* *mean (SD)*	*Low/medium education* *mean (SD)*	*Monday-Friday* *mean (SD)*	*Saturday* + *Sunday* *mean (SD)*
Walking Duration, min/day	128.8 (56.5)	128.8 (53.3)	128.7 (59.6)	132.5 (45.6)	123.5 (47.3)	130.6 (55.2)	124.7 (58.9)
Steps, counts/day	11 746 (5 446)	11 822 (5 194)	11 667 (5 693)	12 078 (4 310)	11 259 (4 546)	11 913 (5 321)	11 381 (5 677)
Number of total WB, counts/day	432.3 (153.8)	453.1 (152.0)	410.9 (152.5)	430.1 (134.4)	434.5 (125.7)	441.4 (153.2)	411.1 (152.5)
Number of longer (> 30 s) WBs, counts/day	44.6 (26.3)	41.5 (24.3)	47.7 (27.9)	42.2 (21.5)	46.8 (21.3)	45.8 (26.1)	41.5 (26.5)
Mean walking speed in longer (> 30 s) WBs, m/s	0.75 (0.13)	0.75 (0.13)	0.76 (0.13)	0.74 (0.11)	0.76 (0.12)	0.76 (0.13)	0.74 (0.14)
P90 walking speed in longer (> 30 s) WBs, m/s	1.01 (0.20)	1.00 (0.20)	1.02 (0.20)	0.98 (0.19)	1.01 (0.20)	1.02 (0.20)	0.99 (0.21)

Presented are digital mobility outcomes (DMOs) for the total sample and stratified by sex, education and the differentiation of weekday (Monday to Friday) and weekend (Saturday + Sunday), as differentiating between working day (Monday to Friday) and weekend (Saturday, Sunday)

*Abbreviations*: interval, *WB* walking bout; P90: 90th percentile

Higher educational attainment was associated with slightly higher levels of walking amount and longer WB (β = 0.08 - 0.09; all *p* ≤ 0.05), whereas no significant associations were observed for living status (p > 0.05, Table S5: Fixed Effects Step1).

### Associations with environmental conditions

While the city of residence was not associated with any DMOs, daylight hours showed small but significant positive associations with both DMOs related to amount (β = 0.07; all *p* ≤ 0.05; Table S5: Fixed Effects Step1). Notably, the association between daylight hours and the number of WB > 30 s was close to level of significance (β = 0.08; *p* = 0.060; Table S5: Fixed Effects Step1).

### Day-to-day variability

**Fig. 2:**
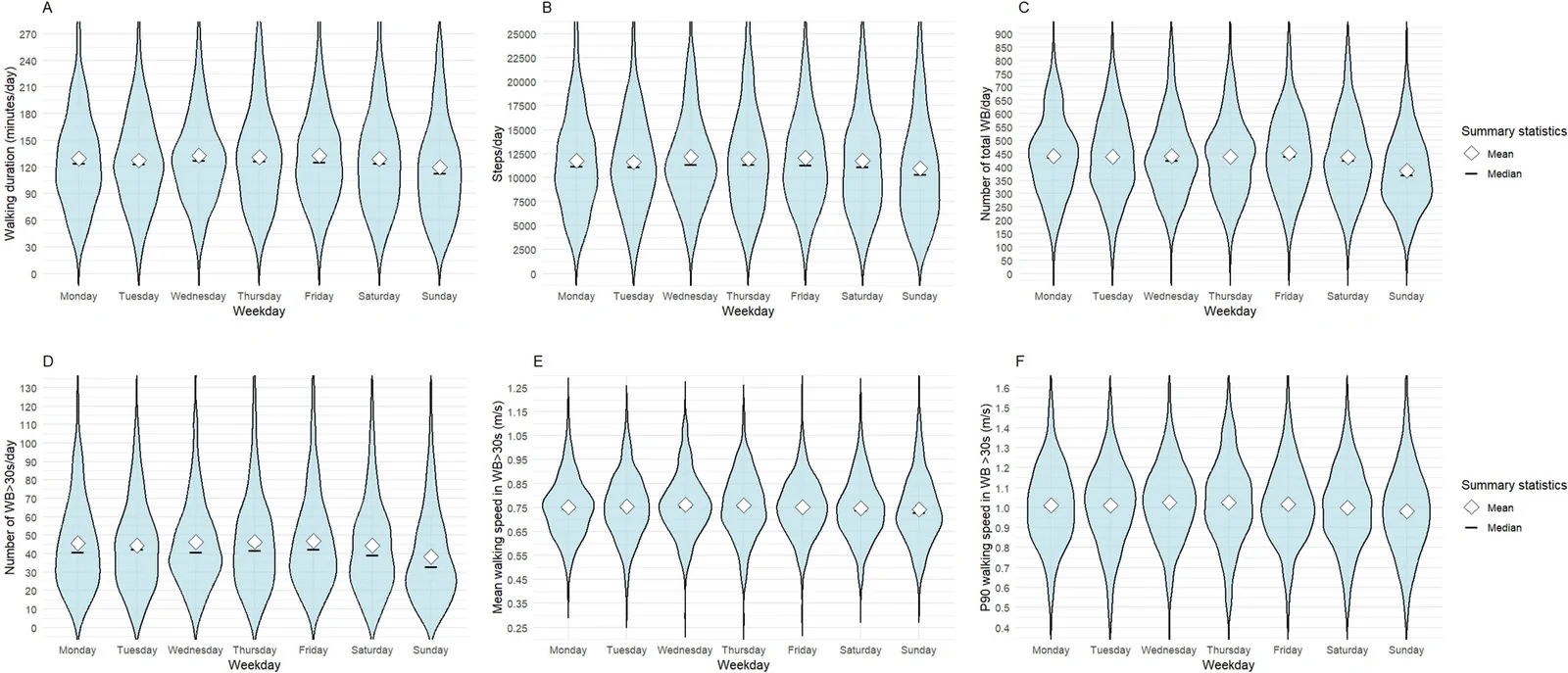
Violin plots depict: **A** Total walking duration in minutes/day; **B** Number of steps/day; **C** Number of total walking bouts (WB)/day; **D** Number of longer (> 30 s) walking bouts/day; **E** Mean walking speed in longer (> 30 s) walking bouts (m/s); **F** 90th percentile (P90) walking speed in longer (> 30 s) walking bouts (m/s) across all weekdays, showing both mean and median values based on raw data

Violin plots based on raw data show largely comparable distributions of DMOs across weekdays, indicating stable activity patterns from Monday to Saturday, while Sundays were characterized by consistently lower levels of real-world walking (Fig. 2 A-F), particularly for longer (> 30 s) WB (Fig. 2 D). Detailed descriptive statistics for all digital mobility outcomes across individual weekdays are reported in Table S7 (Descriptive results by weekday).

In the linear mixed-effect models, weekday-related random-effect variance components were consistently small across all three model stages and all DMOs (Table S8: Random Effects). In contrast, the dichotomous fixed effect distinguishing working days from weekends revealed small and significant differences in the number of WBs > 30 s, mean and P90 walking speed in WBs > 30 s (β = 0.10 to 0.18; all *p* < 0.05; Table S5: Fixed Effects Step1).

### Associations with health and function related outcomes

After adjusting for sociodemographic and environmental characteristics that showed significant associations in the base LMMs, higher locomotor capacity (indicated by lower TUG times) and lower BMI showed consistent significant relations to higher levels of DMOs related to amount, pattern and pace (β = −0.07 to −0.25; all *p* ≤ 0.05). Notably, lower TUG times were more strongly associated with faster mean and P90 walking speed (β =−0.21 to −0.25) than to DMOs representing amount and pattern (β =−0.08 to −0.12) (Table 4).

**Table 4:** Final linear mixed models predicting digital mobility outcomes (DMOs)

Predicting Variables	Outcome											
	*Walking Duration *^*a*^		*Number of Steps *^*b*^		*Number of total WBs *^*c*^		*Number of longer (*> *30 s) WBs *^*d*^		*Mean walking speed in longer (*> *30 s) WBs (m/s) *^*e*^		*P90 walking speed in longer (*> *30 s) WBs (m/s) *^*f*^	
	β (95% CI)	*P*	β (95% CI)	*P*	β (95% CI)	*P*	β (95% CI)	*P*	β (95% CI)	*P*	β (95% CI)	*P*
*BMI [kg/m*^*2*^*]*	−0.22 (−0.29, −0.16)	** < 0.001**	−0.22 (−0.28, −0.15)	** < 0.001**	−0.21 (−0.28, −0.14)	** < 0.001**	−0.17 (−0.24, −0.10)	** < 0.001**	−0.07 (−0.14, 0.01)	**0.035**	−0.17 (−0.23, −0.10)	** < 0.001**
*FCI*	−0.08 (−0.14, −0.01)	**0.018**	−0.08 (−0.14, −0.01)	**0.018**	−0.03 (−0.10, 0.04)	0.416	−0.06 (−0.13, 0.01)	0.163	−0.05 (−0.12, 0.02)	0.155	−0.05 (−0.11, 0.02)	0.155
*TUG [s]*	−0.10 (−0.17, −0.03)	**0.004**	−0.12 (−0.19, −0.05)	**0.001**	−0.11 (−0.19, −0.04)	**0.005**	−0.09 (−0.16, −0.01)	**0.018**	−0.21 (−0.28, −0.14)	** < 0.001**	−0.25 (−0.32, −0.19)	** < 0.001**
*Short FES-I*	−0.09 (−0.16, −0.02)	**0.017**	−0.08 (−0.15, −0.01)	**0.017**	−0.08 (−0.15, −0.01)	**0.032**	−0.08 (−0.16, −0.02)	**0.032**	0.01 (−0.06, 0.08)	0.807	−0.02 (−0.09, 0.04)	0.807
*Symbol Search Score*	0.01 (−0.06, 0.07)	0.862	0.01 (−0.06, 0.08)	0.862	−0.02 (−0.09, 0.05)	0.954	0.01 (−0.07, 0.07)	0.954	0.09 (0.02, 0.16)	**0.016**	0.10 (0.04, 0.17)	**0.005**
*Marginal R*^*2*^	0.13		0.13		0.11		0.11		0.11		0.20	
*Conditional R*^*2*^	0.57		0.55		0.66		0.59		0.60		0.61	
*ICC*	0.58		0.56		0.65		0.59		0.62		0.62	

Presented are standardized regression coefficients (β) with 95% confidence intervals (CI) and p-values from the final linear mixed models of Step 4. All models were adjusted for significant sociodemographic correlates, weekday and environmental predictors identified in Step 1. Marginal R2 represents variance explained by fixed effects; conditional R2 represents variance explained by both fixed and random effects. All p-values are adjusted for multiple comparisons using the Benjamini–Hochberg false discovery rate (FDR) procedure within each mobility domain (amount, pattern, and pace), grouping related DMOs per domain. Standardized coefficients should not be compared directly across DMOs because final adjustment sets differed across outcomes. Intra-Class Coefficients (ICC) were calculated based on unconditional random-intercept models including participant-level clustering

*Abbreviations*: *BMI* Body mass Index, *FCI* Functional Comorbidity Index, *TUG* Timed Up and Go test, *Short FES-I* Short Falls Efficacy Scale International

^a^adjusted for age, education, daylight hours, weekday/weekend ^b^adjusted for age, education, daylight hours, weekday/weekend; ^c^adjusted for age, sex, weekday/weekend; ^d^adjusted for age, sex, education, weekday/weekend; ^e^adjusted for age age2, weekday/weekend, ^f^adjusted for age, age2, sex, weekday/weekend

For all other correlates, we observed heterogeneous associations with DMOs. For example, lower fall-related concerns were weakly but significantly associated with DMOs of amount and pattern (β =—0.08 to −0.09, *p* ≤ 0.05), but not with DMOs of pace (β = −0.01 to −0.02, all *p* ≥ 0.05). In contrast, higher Symbols Search Score showed no significant associations with amount and pattern DMOs (all *p* ≥ 0.05), but was significantly related to higher mean and P90 walking speed (β = 0.09, both *p* ≤  0.020). Marginal R^2^ values ranged from approximately 0.11 to 0.20, indicating moderate proportions of variance explained by the individual correlates included. Intraclass correlation coefficients ranged from 0.56 to 0.65 across the six DMOs, indicating that a substantial proportion of the total variance was attributable to between-participant differences (Table 4).

## Discussion

Overall, this study provides insight into new DMOs representing different aspects of real-world walking, for the first time in a large sample of community-dwelling older adults. The results show a highly functioning and educated sample demonstrating high levels of walking activity, indicating that this is a relatively healthy and actively ageing cohort. Increased age was associated with lower real-world walking across all DMOs, whereas indications of a non-linear relationship with a plateau up to the age of ~ 75 years were not statistically robust for all mobility metrics. Beyond individual characteristics, our findings point to associations with contextual proxies, including day length and weekday versus weekend differences. Real-world walking speed emerged as a key indicator of real-world walking performance, complementing established markers of walking amount.

### Walking activity

In our sample, walking activity clearly exceeded evidence-based step-count recommendations derived from recent cohort studies [45, 46] and meta-analyses corresponding to activity levels associated with a substantially reduced mortality risk [8].

As this study applied a pipeline specifically validated for monitoring daily mobility in older adults, comparisons with studies using different methodologies should be interpreted cautiously. Nevertheless, comparison with recent studies from Norway [23] and Italy [45], which also used the Mobilise-D pipeline in physically robust community-dwelling cohorts, highlights the exceptionally high walking activity of the present sample, reflected in higher step counts as well as more total and longer walking bouts.

### Walking speed

While participants demonstrated high levels of activity, they walked more slowly on average and at their maximum level (P90) than a comparable Norwegian cohort [23]. These findings further underline that the amount of walking, as a behaviour-driven mobility measure, is not necessarily congruent with walking speed, which reflects qualitative and performative aspects of mobility. Nevertheless, P90 walking speed values indicated preserved locomotor capacity relative to established clinical thresholds discriminating between individuals with and without mobility impairments [9, 47].

Importantly, differences between mean and P90 walking speed (0.26 m/s in average) suggest that real-world walking speed captures meaningful intra-individual variability. This variability may reflect adaptive capacity in response to environmental or situational demands, providing information beyond average performance. However, bout-to-bout variability metrics remain exploratory in nature, with mixed evidence regarding their clinical validity and limited interpretability due to the current lack of reference values [33], highlighting the need for further investigation.

It should also be acknowledged that only walking speed was included as a component of gait quality, despite gait quality comprising additional characteristics such as step-to-step gait variability and gait asymmetry, which have been linked to fall risk and mobility impairments [48]. While the present study primarily focused on walking speed-related metrics, future studies should further investigate complementary measures of walking variability in real-world settings.

### Sociodemographic correlates

We observed a consistent age-related decrease of real-world walking across all DMOs. Although visual inspection suggested a possible non-linear pattern with an accelerated decline after the age of 75, as demonstrated previously [46], this trend was only robust for walking speed metrics after adjustment for sociodemographic and environmental factors.

Despite similar overall age-related patterns across sexes, we explored sex-related differences of walking patterns, confirming previous studies conducted in diverse older populations [22]. The finding that women exhibited more WBs in total, but fewer longer WBs than men might be attributed to classical gender roles and generational stereotypes. Mean walking speed did not differ between men and women overall, while small significant differences were found in P90 walking speed.

Even in this highly educated sample, we observed a small effect of educational status on walking activity, but not on walking speed. Interestingly, we found individuals with university degrees to engage more frequently in longer WB than individuals without, possibly indicating higher levels of activities outside the home.

### Health and function related outcomes

Overall, our results demonstrated only weak associations between individual characteristics of locomotor capacity, psychosocial function, and cognitive processing speed with metrics of real-world walking. Lower TUG times and lower BMI were the most consistent correlates of DMOs, although associations remained weak, consistent with previous findings in physically robust older adults [49, 50]. This is consistent with the conceptual distinction between capacity and performance, highlighting that what individuals are physically capable of (“can do”) is only partly reflected in their everyday mobility behaviour (“does do”).

Participants also demonstrated a high Symbol Search Score, indicating preserved cognitive processing speed. While cognitive processing speed was not linked to the amount and patterns of walking activity, it was significantly associated with real-world walking speed, in line with meta-analytic evidence linking cognition and supervised gait speed [51]. Considering that gait speed assessed under laboratory conditions primarily represents a single-task paradigm, whereas real-world walking involves additional cognitive demands [11], it may be hypothesized that real-world walking speed is even more sensitive to potential cognitive changes.

Overall, real-world walking in individuals without major physical or cognitive limitations may be strongly linked to situational and environmental factors. This is reflected by the large proportion of variance explained by the individual as a random factor, indicating substantial interindividual variability.

### Day-to-day variability

An important component of intraindividual variability is day-to-day variability, which has received limited attention to date. Our results indicated reductions across all domains of real-world walking during weekends, as the sample showed lower amount, decreased pattern and slower pace.

Descriptively, Sundays appeared to contribute most strongly to this pattern, potentially acting as ‘rest days’. This observation aligns with previous findings in German community-dwelling older adults [52], and suggests that walking activity may partly reflect the execution of daily routines, such as shopping or medical appointments, which are generally restricted on Sundays in Germany. These differences at the group level probably indicate even greater differences at the level of individual persons. The level of intra-individual day-to-day variability may provide information about the discrepancy between the maximum possible and the usual activity level. Based on this information, interventions to promote activity can be tailored even better to individual needs of the person.

### Effect of environmental conditions

We found differentiated associations with proxies of environmental conditions on mobility. It was shown that the duration of daylight as a crude proxy of season affected the number of longer WBs, predominantly representing mobility outside the home. This is in line with meta-analytic evidence showing outdoor mobility to be reduced in shorter days and/or during colder weather conditions across all age groups [53].

The results provide initial evidence that the seasonal daylight duration is also associated with the pace of walking, as P90 walking speed was reduced during winter days. This result contradicts findings of a longitudinal study conducted within Japanese adults of various age groups, showing significantly higher walking speeds in colder conditions compared to summertime [54]. These findings may also reflect a potentially non-linear relationship between weather conditions and real-world walking, underscoring the need for more detailed investigations into the interplay between environmental characteristics, weather conditions, and walking patterns. We included the place of residence as a proxy of location with low granularity and we did not observe differences in DMOs between the two cities of Heidelberg and Mannheim. But a recent study revealed the strong impact of built environment on the amount of walking in adults across all ages [55]. This finding requires closer examination using specific walkability characteristics and high spatial resolution in populations of older adults.

### Strengths and limitations

A major strength of this study lies in its ability to characterize real-world walking in a large cohort of older adults using a comprehensive set of validated digital mobility outcomes derived from an open-source data pipeline. We provide transparent and reproducible benchmarks for walking performance in community-dwelling older adults, facilitating comparability for future digital mobility research in other older populations. The study provides differentiated insights into age-related patterns of real-world walking and indicators of gait quality such as walking speed. However, the generalizability of our study findings is limited, as the present sample consists of physically robust, highly active, well-educated, and cognitively unimpaired older adults.

By applying hierarchical linear mixed models, we identified sociodemographic, environmental and modifiable individual correlates that differentially affect distinct domains of walking, underscoring its multifactorial nature. Although the observed associations were generally weak to moderate in magnitude, the statistical inference was strengthened by the application of correction procedures for multiple comparisons. This set of correlates was not exhaustive, as only a limited range of representative individual and environmental markers were included, and potentially relevant interaction effects between the included correlates were not examined to limit model complexity. Other potentially relevant factors (e.g., minor employment, volunteering, frequency of social contacts) may not have been captured. In addition, environmental conditions were operationalized using proxy measures.

Although predictor selection was guided by a priori theoretical considerations, the hierarchical retention approach may still be susceptible to some limitations associated with stepwise modelling procedures, including potential model instability and overfitting. Therefore, the identified correlates should be interpreted cautiously and primarily as exploratory associations.

These findings nevertheless inform the development of more individualized intervention strategies targeting specific mobility domains. Additionally, the observed day-to-day variability in walking has important methodological implications for defining appropriate monitoring periods in studies of real-world mobility. But, due to the exploratory nature of this study, these findings are primarily descriptive and were not subjected to formal inferential statistical tests, and should therefore be interpreted with caution. Further studies are needed to test the temporal variability of walking in older populations.

The Mobilise-D computational pipeline captures only walking-related DMOs, whereas other relevant forms of mobility in active older adults, such as cycling or e-biking [56], are not identified. Furthermore, while gait asymmetry represents a potentially clinically relevant marker of gait quality [57], its valid assessment in real-world conditions using a single-sensor setup remains challenging and warrants further investigation.

## Conclusion

The assessment of real-world walking in older adults using digital technologies allows a multidimensional characterization of mobility that extends beyond amount-based measures. Digital markers of daily-life gait provide additional insights into age- and sex-related walking patterns as well as temporal patterns in real-world walking. Integrating such measures into clinical and research settings may contribute to the understanding of mobility decline in older adults and support more targeted approaches to mobility assessment and mobility-enhancing interventions.

## Supplementary Information

Supplementary Material 1.

## Data Availability

Data from the SMART-AGE study will be published on heiDATA (https://heidata.uni-heidelberg.de), an open research data repository, hosted by Heidelberg University, in summer/autumn 2026. Algorithms to identify walking events are available from the official Mobilise-D repository at https://github.com/mobilise-d/. Codes for statistical analysis is available upon reasonable request.
