# Supplementary material for: Real-world walking and activity patterns in community-dwelling older adults: a cross-sectional analysis of baseline data from the SMART-AGE trial
Source: Eur Rev Aging Phys Act. 2026 Aug 5;23:31. doi: 10.1186/s11556-026-00431-z (PMC13501678; doi:10.1186/s11556-026-00431-z)
Supplement: Supplementary file 1 [file 11556_2026_431_MOESM1_ESM.docx]

# Supplementary Materials

**Table S1**: The total of 24 of Digital Mobility Outcomes (DMOs) extracted by MobGap v1.0 pipeline

|  | Parameter | Unit | Daily calculation |
| --- | --- | --- | --- |
| *Walking*  *Activity* | *Amount* | | |
|  | **Walking duration** | **minutes/day** | **Daily sum of WB duration using all WB** |
|  | **Number of steps** | **#/day** | **Sum of steps using all WB** |
|  | *Pattern* | | |
|  | **Number of WB** | **#/day** | **Daily sum of all WB** |
|  | Number of WB > 10s | #/day | Daily sum of number of WB longer than 10s |
|  | **Number of WB > 30s** | **#/day** | **Daily sum of number of WB longer than 30s** |
|  | Number of WB > 60s | #/day | Daily sum of number of WB longer than 60s |
|  | WB duration | Sec | Daily median of WB duration using all WB |
|  | P90 WB duration | Sec | Daily P90 of WB duration using all WB |
|  | WB duration bout to bout variability | - | Daily COV of WB duration using all WB |
| *Gait Quality* | *Pace* | | |
|  | Walking speed in shorter (10-30s) WB | m/s | Daily mean of walking speed using WB between 10s and 30s |
|  | **Walking speed in longer (>30s) WB** | **m/s** | **Daily mean of walking speed using WB longer than 30s** |
|  | P90 walking speed in WB > 10 s | m/s | Daily P90 of walking speed using WB longer than 10s |
|  | **P90 walking speed in longer (>30s) WB** | **m/s** | **Daily P90 of walking speed using WB longer than 30s** |
|  | Stride length in shorter (10-30s) WB | Cm | Daily mean of stride length using WB between 10s and 30s |
|  | Stride length in longer (>30s) WB | Cm | Daily mean of stride length using WB longer than 30s |
|  | *Rhythm* | | |
|  | Cadence in all WB | steps/min | Daily mean of cadence using all WB |
|  | Cadence in longer (>30s) WB | steps/min | Daily mean of cadence using WB longer than 30s |
|  | P90 cadence in longer (>30s) WB | steps/min | Daily P90 using WB longer than 30s |
|  | Stride duration in all WB | Sec | Daily mean of stride duration using all WB |
|  | Stride duration in longer (>30s) WB | Sec | Daily mean of stride duration using WB longer than 30s |
|  | *Bout-to-Bout Variability* | | |
|  | Walking speed bout-to-bout-variability in longer (>30s) WBs | % | Daily COV of walking speed using WB longer than 30s |
|  | Stride length bout to bout variability in longer (>30s) WBs | % | Daily COV of stride length using WB longer than 30s |
|  | Cadence bout to bout variability | % | Daily COV using all WB |
|  | Stride duration bout to bout variability | % | Daily COV of stride duration using all WB |

Notes: Presented are the 24 DMOs Note: bold letters represent DMOs shown in the present study.

Abbreviations: WB, Walking Bout; P90:,90^th^ Percentile; COV, Coefficient of Variation

**Table S2**: Descriptive results of all 24 Digital Mobility Outcomes (DMOs)

| **DMO** | **Mean (SD)** | **Md (P25 - P75)** |
| --- | --- | --- |
| **Amount** | | |
| Walking duration, min/day | 128.8 (56.5) | 124.1 (88.2 - 161.2) |
| Number of steps, counts/day | 11 746 (5 446) | 11 129 (7 834 – 14 814) |
| ***Pattern*** | | |
| Number of WBs, counts/day | 432.3 (153.8) | 426.0 (322.0 - 527.0) |
| Number of WB>10s, counts/day | 207.2 (79.7) | 200.0 (150.0 - 255.0) |
| Number of WB >30s, counts/day | 44.5 (26.3) | 40.0 (26.0 - 58.0) |
| Number of WB>60s, counts/day | 14.5 (11.8) | 12.0 (6.0 - 20.0) |
| WB duration, s | 9.8 (1.4) | 9.5 (8.9 - 10.6) |
| P90 WBs duration, s | 31.5 (11.8) | 29.2 (25.0 - 34.6) |
| WBs duration bout-to-bout variability | 177.2 (97.8) | 155.9 (105.5 - 219.0) |
| ***Pace*** | | |
| Walking speed in shorter (10-30s) WBs, m/s | 0.60 (0.07) | 0.60 (0.55 - 0.64) |
| Walking speed in longer (>30s) WBs, m/s | 0.75 (0.13) | 0.75 (0.67 - 0.83) |
| P90 walking speed in WBs (>10s), m/s | 0.87 (0.15) | 0.87 (0.78 - 0.95) |
| P90 walking speed in longer (>30 s) WBs, m/s | 1.01 (0.20) | 1.01 (0.88 - 1.14) |
| Stride length in shorter (10–30 s) WBs, m | 0.80 (0.08) | 0.80 (0.75 - 0.85) |
| Stride length in longer (>30 s) WBs, cm | 0.96 (0.13) | 0.95 (0.87 - 1.04) |
| **Rhythm** | | |
| Cadence in all WBs, steps/min | 86.2 (3.9) | 86.0 (83.6 - 88.8) |
| Cadence in longer (>30s) WBs, steps/min | 92.0 (6.1) | 91.7 (87.9 - 95.8) |
| P90 cadence in longer (>30s) WBs, steps/min | 103.2 (8.2) | 103.0 (97.6 - 109.0) |
| Mean stride duration in all WB, s | 1.27 (0.06) | 1.27 (1.23 - 1.31) |
| Stride duration in longer (>30 s) WBs, s | 1.23 (0.08) | 1.23 (1.18 - 1.28) |
| ***Variability*** | | |
| Walking speed bout-to-bout variability in longer (>30 s) WBs | 26.3 (7.6) | 26.2 (21.4 - 31.0) |
| Stride length bout-to-bout variability in longer (>30 s) WBs | 19.6 (5.9) | 19.3 (15.8 - 23.1) |
| Cadence bout-to-bout variability | 12.6 (1.5) | 12.6 (11.6 - 13.5) |
| Stride duration bout-to-bout variability | 15.9 (2.1) | 15.7 (14.5 - 16.9) |

**Table S3.** Reasons for exclusion from data analyses

| **Reason** | **Number** |
| --- | --- |
| Technical failure (missing gyroscope data, flat data) | n=35 |
| Early removal/insufficient wear-time for unknown reasons | n=24 |
| Early removal due medical reasons  Allergy  Surgical procedure during measurement period | n=4  n=3  n=1 |
| Missing data sets for unknown reasons | n=8 |
| Failure of initialization/upload | n=3 |
| Denial of wearing the sensor | n=3 |
| Loss of sensor | n=1 |
| Exclusion for analysis due,medical reasons  Parkinson’s Disease  Not able,to walk/wheelchair user | n=2  n=1  n=1 |

| **Variable** | **n** | **%** |
| --- | --- | --- |
| Total walking duration ^a^ | 0 | 0 |
| Number of steps, counts/day  ^a^ | 0 | 0 |
| Number of WBs, counts/day  ^a^ | 0 | 0 |
| Number of WB >30s, counts/day  ^a^ | 0 | 0 |
| Walking speed in longer (>30s) WBs, m/s  ^a^ | 12 | 0.4 |
| P90 walking speed in longer (>30 s) WBs, m/s  ^a^ | 12 | 0.4 |
| Age ^b^ | 0 | 0 |
| Sex  ^b^ | 0 | 0 |
| Education  ^b^ | 0 | 0 |
| Living status  ^b^ | 27 | 4.7 |
| Body Mass Index  ^b^ | 2 | 0.4 |
| Functional Comorbidity Index  ^b^ | 8 | 1.4 |
| Timed Up & Go time  ^b^ | 3 | 0.5 |
| Short-FES-I sum score  ^b^ | 29 | 5.1 |
| Symbol Search Score  ^b^ | 0 | 0 |

**Table S4**. Missing data of variables included in the analyses, expressed as total numbers and percentage

Notes: ^a^ The number of missing data for digital mobility outcomes represent the number of days missing, as the analyses of DMOs were conducted on the daily level. ^b^ Missing data for all other variables, conducted during home visits and via self-assessments refer to the total number of participants included in the final analyses (having valid sensor measurements, n=569)

**Table S5.** Fixed effects of socio-demographic indicators, weekday and environmental conditions on Digital Mobility Outcomes (DMOs)

| **Predicting Variables** | ***Outcomes*** | | | | | | | | | | | |
| --- | --- | --- | --- | --- | --- | --- | --- | --- | --- | --- | --- | --- |
|  | *Walking Duration, minutes/day* | | *Number of steps, counts/day* | | *Number of total WBs, counts/day* | | *Number of longer (>30s) WBs, counts/day* | | *Mean walking speed longer (>30s) in WB, m/s ^a^* | | *P90 walking speed in longer (>30s) WBs, m/s* | |
|  | $\beta$*(95% CI)* | *p* | $\beta$*(95% CI)* | *p* | $\beta$*(95% CI)* | *p* | $\beta$*(95% CI)* | *p* | $\beta$*(95% CI)* | *p* | $\beta$*(95% CI)* | *p* |
| *Age* | -0.13  (-0.20, -0.06) | **<0.001** | -0.14  (-0.21, -0.07) | **0.001** | -0.10  (-0.17, -0.02) | **0.008** | -0.11  (-0.18, -0.04) | **0.003** | -0.23  (-0.30, -0.16) | **<0.001** | -0.26  (-0.33, -0.19) | **<0.001** |
| *Sex [male]* | -0.06  (-0.20, 0.09) | 0.449 | -0.07  (-0.22, 0.07) | 0.449 | -0.29  (-0.44, -0.14) | **<0.001** | 0.17  (0.03, 0.32) | **0.018** | 0.12  (-0.03, 0.26) | 0.093 | 0.20  (0.06, 0.35) | **0.012** |
| *Education (high)* | 0.09  (0.02, 0.16) | **0.010** | 0.09 (0.02,0.16) | **0.010** | 0.02  (-0.05, 0.10) | 0.548 | 0.08  (0.01, 0.15) | **0.048** | 0.00  (-0.08, 0.06) | 0.796 | 0.01  (-0.06, 0.08) | 0.796 |
| *Living Status (with partner)* | -0.04  (-0.11, 0.19) | 0.653 | 0.03  (-0.11, 0.18) | 0.653 | 0.10  (-0.06, 0.25) | 0.220 | 0.12  (-0.03, 0.27) | 0.209 | -0.10  (-0.25, 0.05) | 0.254 | -0.09  (-0.24, 0.06) | 0.254 |
| *Weekday (weekend)* | -0.12  (-0.22, -0.02) | **0.041** | -0.11  (-0.20, -0.02) | **0.041** | -0.21  (-0.39, -0.03) | **0.037** | -0.18  (-0.30, -0.06) | **0.028** | -0.09  (-0.14, -0.05) | **<0.001** | -0.15  (-0.23, -0.07) | **0.005** |
| *Daylight hours* | 0.07  (0.00, 0.14) | **0.045** | 0.07  (0.00, 0.14 | **0.045** | 0.03  (-0.05, 0.10) | 0.459 | 0.08  (0.01, 0.15) | 0.060 | -0.02  (-0.09,0.05) | 0.557 | -0.07  (-0.14, 0.00) | 0.101 |
| *City (Mannheim)* | -0.02  (-0.16, 0.12) | 0.915 | -0.00  (-0.15, 0.13) | 0.915 | 0.07  (-0.07, 0.22) | 0.615 | -0.02  (-0.16, 0.12) | 0.740 | 0.03  (-0.11, 0.17) | 0.686 | 0.03  (-0.11, 0.17) | 0.686 |
| *Marginal R²* | 0.03 | | 0.03 | | 0.04 | | 0.05 | | 0.05 | | 0.08 | |
| *Conditional R²* | 0.56 | | 0.56 | | 0.66 | | 0.59 | | 0.61 | | 0.63 | |

Notes: Presented are standardized Beta-Coefficients, 95% Confidence Intervals (95% CI) and *p*-values based on Linear Mixed Models (LMMs). *P*-values <.05 are written in bold numbers. Notes: a For the LMM, predict mean walking speed in WB>30s, weekday was deleted as random factor, as the model showed singularity and random-effect variance of weekday was estimated at zero. For the dichotomous variables of sex, living status, weekday/weekend and City the reference categories are written in brackets.

**Table S6**. Model fit comparison of linear and quadratic age effects for digital mobility outcomes

| Model Fit | Walking Duration | Number of steps | Number of total WB | Number of WB>30s | Mean walking speed in WB >30s | P90 walking speed in WB >30s |
| --- | --- | --- | --- | --- | --- | --- |
| AIC (linear age term) | 33092.3 | 62232.9 | 38935.4 | 28159.9 | -5612.7 | -2901.5 |
| AIC (quadratic age term) | 33092.7 | 62232.8 | 38936.8 | 28161.6 | -5616.8 | -2903.6 |
| ΔAIC | 0.4 | -0.2 | 1.4 | 1.7 | -4.0 | -2.1 |
| BIC (linear age term) | 33159.0 | 62299.7 | 39002.2 | 28226.6 | -5552.1 | -2834.8 |
| BIC (quadratic age term) | 33165.5 | 62305.6 | 39009.6 | 28234.4 | -5550.1 | -2830.8 |
| ΔBIC | 6.5 | 5.9 | 7.4 | 7.8 | 2.1 | 4.0 |
| χ² | 1.6 | 2.2 | 0.6 | 0.3 | 6.0 | 4.1 |
| Df | 1 | 1 | 1 | 1 | 1 | 1 |
| p-value | 0.207 | 0.142 | 0.423 | 0.577 | **0.014** | **0.043** |
| LRT Model preference | linear | linear | linear | linear | quadratic | quadratic |
| AIC model preference | linear | quadratic | linear | linear | quadratic | quadratic |
| BIC model preference | linear | linear | linear | linear | linear | linear |

Notes: Presented are the model fit comparison between linear and quadratic age specifications for each digital mobility outcome. Lower AIC/BIC values and the χ² difference tests reflect whether adding the quadratic age term significantly improved model fit. Significant χ² values indicate that the quadratic specification provided a better fit than the linear model, whereas non-significant χ² tests suggest that the linear age term was sufficient. Based on that we report model preference. LRT, Likelihood-Ratio-Test; AIC, Aikake Information Criterion; ΔAIC, difference in Akaike Information Criterion between quadratic age term model and base model; BIC, Bayesian Information Criterion; ΔBIC, difference of Bayesian Information Criterion between quadratic age term model and base model; χ²= Chi²; WB: walking bout

**Table S7.** Descriptive results of Digital Mobility Outcomes (DMOs) differentiated by weekday

| **weekday** | *Walking duration, minutes/day* | | *Number of steps,*  *counts/day* | | *Number of total WBs counts/day* | | *Number of longer (>30s) WBs, counts/day* | | *Mean walking speed longer (>30s) in WBs, m/s* | | *P90 walking speed in longer (>30s) WB, m/s* | |
| --- | --- | --- | --- | --- | --- | --- | --- | --- | --- | --- | --- | --- |
|  | *Mean*  *SD* | *Md*  *(P25-P75)* | *Mean (SD)* | *Md*  *(P25 - P75)* | *Mean (SD)* | *Md*  *(P25 - P75)* | *Mean*  *(SD)* | *Md*  *(P25 -P75)* | *Mean*  *SD* | *Md*  *P25 - P75* | *Mean*  *SD* | *Md*  *(P25 - P75)* |
| Monday | 129.4 (56.1) | 124.4  (91.0 - 162.0) | 11 760 (5 362) | 11 194  (8 059 – 14 784) | 440.3  (151.7) | 436.5  (330.8 - 529.0) | 45.6  (26.7) | 41.0  (26.8 - 60.0) | 0.75 (0.13) | 0.75  (0.66 - 0.82) | 1.01  0.20 | 1.01  (0.87 - 1.15) |
| Tuesday | 127.3 (52.7) | 123.5  (89.0 - 160.8) | 11 608 (5 075) | 11 151  (7 960 - 14 744) | 439.0  (155.5) | 436.5  (335.8 - 550.0) | 44.4  (24.1) | 42.5  (26.8 - 58.0) | 0.75 (0.13) | 0.75  (0.67 - 0.84) | 1.01  0.20 | 1.02  (0.88 - 1.14) |
| Wednesday | 132.8 (54.9) | 127.4  (96.8 - 159.9) | 12 167 (5 336) | 11 402  (8 753 – 14 817) | 440.0  (153.0) | 426.0  (338.0 - 524.0) | 46.3  (25.9) | 41.0  (29.0 - 58.0) | 0.76 (0.13) | 0.76  (0.68 - 0.83) | 1.03  0.19 | 1.03  (0.91 - 1.15) |
| Thursday | 130.8 (55.7) | 127.1  (88.8 - 164.1) | 11 955 (5 381) | 11 413  (7 850 – 15 110) | 437.8  (152.7) | 442.0  (327.0 - 524.0) | 46.5  (27.1) | 42.0  (27.0 - 60.0) | 0.76 (0.13) | 0.76  (0.68 - 0.85) | 1.03  0.22 | 1.02  (0.90 - 1.15) |
| Friday | 132.8 (56.5) | 125.8  (92.8 - 162.5) | 12 082 (5 439) | 11 309  (8 241 – 14 971) | 452.3  (154.0) | 441.5  (348.8 - 555.3) | 46.8  (26.5) | 42.5  (28.0 - 60.0) | 0.75 (0.12) | 0.75  (0.67 - 0.83) | 1.02  0.20 | 1.01  (0.89 - 1.14) |
| Saturday | 129.2 (58.7) | 124.2  (86.3 - 164.1 | 11 751 (5 607) | 11 138  (7 538 – 14 909) | 436.4  (158.1) | 424.5  (326.8 - 536.5) | 44.5  (27.4) | 39.5  (25.0 - 58.0) | 0.75 0.13) | 0.74  (0.66 - 0.83) | 1.00  0.20 | 1.00  (0.88 - 1.13) |
| Sunday | 119.9 (58.9) | 113.1  (76.1 - 152.6) | 10 991 (5 735) | 10 347  (6 703 – 14 060) | 385.8  (143.0) | 370.5  (282.8 - 474.3) | 38.5  (25.3) | 33.0  (20.0 - 52.0) | 0.74 (0.14) | 0.73  (0.65 - 0.82) | 0.98  0.21 | 0.98  (0.84 - 1.11) |

Presented are mean values, standard deviations (SD), median values and 25^th^ (P25) and 75^th^ Percentile (P75).

**Table S8.** Random-effect variance components for all steps of the hierarchical linear mixed models (LMMs) predicting daily mobility outcomes.

| **DMO** | **Random Effect Variance (SD)** | **Hierarchical Model Step** | | |
| --- | --- | --- | --- | --- |
|  |  | *Base Model* | *Quadratic Age Term* | *Final Model* |
| **Walking Duration,** | *Participant* | 1664.0 (40.8) | 1658.0 (40.7) | 1389.8 (37.3) |
|  | *Weekday* | 6.3 (2.5) | 6.3 (2.5) | 6.7 (2.6) |
|  | *Residual* | 1333.2 (36.5) | 1333.3 (36.5) | 1368.9 (37.0) |
| **Number of steps** | *Participant* | 14941956 (3866) | 14869277 (3856) | 12418340 (3524) |
|  | *Weekday* | 46593 (216) | 46570 (216) | 46782 (216) |
|  | *Residual* | 12733997 (3569) | 12734456 (3569) | 13155024 (3627) |
| **Number of total WBs** | *Participant* | 13939 (118.1) | 13919 (118.0) | 12590 (112.2) |
|  | *Weekday* | 196.7 (14.0) | 196.7 (14.0) | 212.1 (14.6) |
|  | *Residual* | 7920.7 (89.0) | 7920.8 (89.0) | 8007.5 (89.5) |
| **Number of longer (>30s) WBs** | *Participant* | 364.7 (19.7) | 364.4 (19.1) | 333.4 (18.3) |
|  | *Weekday* | 2.3 (1.5) | 2.3 (1.5) | 2.5 (1.6) |
|  | *Residual* | 281.3 (16.8) | 281.3 (16.8) | 285.9 (16.9) |
| **Mean walking speed in longer (>30s) WBs** | *Participant* | 0.009 (0.09) | 0.010 (0.098) | 0.008 (0.090) |
|  | *Weekday* | a | a | a |
|  | *Residual* | 0.007 (0.08) | 0.007 (0.082) | 0.007 (0.082) |
| **P90 walking speed in longer (>30s) WBs** | *Participant* | 0.023 (0.15) | 0.023 (0.151) | 0.016 (0.128) |
|  | *Weekday* | 0.001 (0.06) | b | b |
|  | *Residual* | 0.016 (0.12) | 0.016 (0.127) | 0.016 (0.126) |

Notes: Presented are variances for the random effects of the individual participant, weekday and residuals for each of the hierarchical linear model steps. a For all models of mean walking speed in WB>30s random variances were estimated as zero, indicating no detectable between-weekday variability in this outcome. Therefore, no random-effect variance for weekday could be reported for this model. ^b^ Random-effect variance estimates for the quadratic age term model and the final model of P90 walking speed are not presented because the estimated variances were negligible (i.e., below the fourth decimal place). Abbreviations: WB, Walking Bouts; P90, 90^th^ Percentile; SD, Standard Deviation.

**Figure S1**: Histograms of Digital Mobility Outcomes (DMOs)

Notes: Presented are histograms for daily walking duration, number of steps, number of total walking bouts (WB), number of walking bouts >30s, mean walking speed >30s, Percentile 90 walking speed >30s

**Figure S2**: Q–Q Plots of Residuals from the Final Linear Mixed-Effects Models Across Six Digital Mobility Outcomes

Notes: Q–Q plots of residuals for the final models of (A) walking duration, (B) number of steps, (C) total number of walking bouts (WB), (D) number of longer walking bouts (>30 s), (E) mean walking speed in longer walking bouts (>30 s), and (F) P90 walking speed in longer walking bouts (>30 s). Overall, residuals showed approximate normality with minor deviations in the distribution tails, indicating slight departures from normality particularly for extreme values.
